# Improvement of signal-to-noise ratio in parallel neuron arrays with spatially nearest neighbor correlated noise

**DOI:** 10.1371/journal.pone.0200890

**Published:** 2018-07-18

**Authors:** Tianquan Feng, Qingrong Chen, Ming Yi, Zhongdang Xiao

**Affiliations:** 1 College of Teacher Education, Nanjing Normal University, Nanjing, China; 2 State Key Laboratory of Bioelectronics, School of Biological Science and Medical Engineering, Southeast University, Nanjing, China; 3 School of Psychology, Nanjing Normal University, Nanjing, China; 4 College of Sciences, Huazhong Agricultural University, Wuhan, China; Universitat Pompeu Fabra, SPAIN

## Abstract

We theoretically investigate the signal-to-noise ratio (SNR) of a parallel array of leaky integrate-and-fire (LIF) neurons that receives a weak periodic signal and uses spatially nearest neighbor correlated noise. By using linear response theory, we derive the analytic expression of the SNR. The results show that the amplitude of internal noise can be increased up to an optimal value, which corresponds to a maximum SNR. Given the existence of spatially nearest neighbor correlated noise in the neural ensemble, the SNR gain of the collective ensemble response can exceed unity, especially for a negative correlation. This nonlinear collective phenomenon of SNR gain amplification may be related to the array stochastic resonance. In addition, we show that the SNR can be improved by varying the number of neurons, frequency, and amplitude of the weak periodic signal. We expect that this investigation will be useful for both controlling the collective response of neurons and enhancing weak signal transmission.

## Introduction

Stochastic resonance (SR) was first introduced in the context of worldwide climatic changes [1]. This mechanism has potential applications in the field of the complex signal processing as it can be used to extract weak signals from a noisy background. In fact, signals that previously would have been considered lost can be detected and retrieved using SR-based methods. SR is usually defined from measures such as the output signal-to-noise ratio (SNR) of a nonlinear system with periodic input, where the SNR shows a nonmonotonic trend with respect to the level of background noise. Moreover, SR has been observed in different systems [2–5], including lasers, sets of neurons, solid-state devices, among others [6–11]. Likewise, extensive research has been devoted to investigate the output SNR gain with respect to an input SNR for periodic signals and Gaussian noise in nonlinear systems [12–15].

SR investigation has also attracted attention in neuroscience, given the abundance of noisy signals and related effects. For instance, in some organisms, periodic signals are transformed from external stimuli into spike trains via sensory neurons in the nervous system. This fact and the inclusion of noise have motivated many studies on peripheral sensory systems that exhibit SR. In addition, SR has been found in various neural models and related experimental observations [4, 12–22]. Furthermore, there are several studies on SR considering both a single neuron and parallel arrays of neurons, where the latter have been investigated to assess collective responses and the possibility of SNR gain [12–15]. However, unlike a single neuron system, the structure of neural ensembles and noise sources increase complexity and variability. Hence, investigations of SR and SNR gain in parallel neuron arrays considering noise may lead to the discovery of novel phenomena.

For instance, the results in [5] demonstrate that neural ensembles can reliably detect subthreshold pulses by injecting appropriate noise. Recently, SNR improvement induced by internal noise has been achieved in a parallel array including several nonlinear subsystems [23–26]. The noise effect in such arrays reveals important phenomena different from the conventional SR, such as SR without tune [20], suprathreshold SR [15] and array SR [23]. All the subsystems in these arrays share a single input signal, and the outputs are generated by their collective response [23–26]. Given that the nervous systems of different organisms, including humans, have a complex structure with a variety of neurons with specific functions and sizes, research on the noise effect of neuron arrays in the SR and SNR is still an ongoing endeavor [27], especially when considering the threshold-based sensory neuron model. Nevertheless, despite the abundant research on the role of internal and external noise, it is still needed to clarify the variability mechanisms in noise sources of complex neural networks, the optimization of noise background, and the correlation among internal noise by using nonlinear subsystems [17].

In fact, research on neural ensembles has been mainly focused on the independent internal noise of each neuron [23–26]. In contrast, we aim to unveil the correlation of noise in a neural ensemble. Previous work showed that correlated noise mitigates the general noise effects [23], and we found that the spatially nearest neighbor correlation among neurons can be generalized for a neural ensemble. In other words, the spatially nearest neighbor correlation among neurons is more beneficial for the ensemble when considering weak periodic signals. Hence, we examine the effect of spatially nearest neighbor correlation among neurons on the SR from a parallel array of leaky integrate-and-fire (LIF) neurons. The correlation of noise can be considered as a more realistic assessment of SR in the brain [28–33].

**Fig 1:**
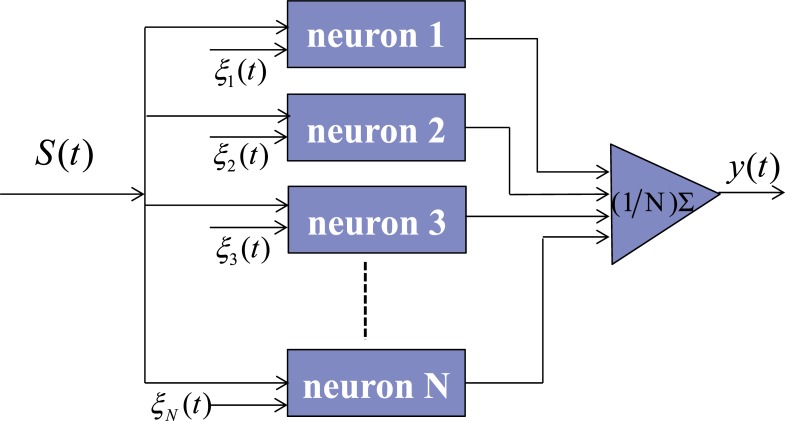
Parallel array of *N* LIF neurons. Every neuron receives the same input signal, *S*(*t*), and white Gaussian noise *ξ*_*i*_(*t*) (1 ≤ *i* ≤ *N*) characterizes the internal stochastic process of the *i*-th neuron. The average spike train, $y(t)=\frac{1}{N}\sum_{i=1}^{N}y_{i}(t)$, is the array output.

In this article, we consider an LIF neuron array with *N* neurons, as illustrated in Fig 1, because this model is widely used to study the nonlinear dynamics of neural systems. Moreover, it can provide an exact description of the subthreshold integration of synaptic inputs. Hence, by assembling LIF neurons into an array, we show that the collective response to a weak periodic signal with noise can be improved by the internal noise of each neuron. Furthermore, we derive and analyze the SNR gain for this type of signal. Extended theoretical investigations considering other neural models (e.g., the Hodgkin–Huxley and FitzHugh–Nagumo models) with different types of noises [34, 35] is beyond the scope of this article.

## Methods

### Parallel array of LIF neurons

Let us consider a parallel array of *N* LIF neurons driven by the input *I*_*i*_(t), as illustrated in Fig 1. The *i*-th LIF neuron is described by membrane potential *V*_*i*_, which is governed by the following equation [25, 26]:

$$\frac{dV_{i}(t)}{dt}=-V_{i}(t)+I_{i}(t)$$ (1)

with1 *≤ i ≤ N*. Here time is measured in units of the membrane time constant, the resistance of the cell membrane is lumped into the input, and the voltage variable and input are rescaled by a typical value such that all variables and parameters are nondimensional. The threshold–spike–reset condition states that a spike will be generated whenever the membrane potential satisfies *V*_*i*_(*t*) = *V*_th_. Then, the membrane potential will be maintained during refractory period *τ*_r_. In the following we set *V*_th_ = 1 and *V*_r_ = 0. The output spike train of each LIF neuron is determined by *y*_*i*_(*t*) = Σ_*k*_ *δ*(*t − t*_*i*_^*k*^), with *t*_*i*_^*k*^ being the instant when the *k*-th spike occurs.

The input *I*_*i*_ (t) is given by

$$I_{i}(t)=\mu +\xi_{i}(t)+S(t),$$ (2)

where *μ* is the constant base current. Note that, in this article we choose a small bias current *μ <* 1 such that the neurons are in a subthreshold firing regime, i.e., spikes are induced by internal or external noise. The internal noise processes *ξ*_*i*_(*t*) represent the internal stochastic dynamics of the *i*-th neuron. The internal noise term *ξ*_*i*_(*t*) are zero-mean Gaussian white noise of intensity *D*, i.e.,

$$\langle \xi_{i}(t)\rangle =0,\langle \xi_{i}(t)\xi_{j}(t+\tau )\rangle =2D[\delta_{i,j}+\lambda \delta_{i,j-1}+\lambda \delta_{i,j+1}]\delta (\tau ),$$ (3)

where 1 *≤ i*, *j ≤ N* and |*λ*|≤1 with *λ* a tunable spatially nearest neighbor correlation coefficient. For *λ* = 0 all internal noise is uncorrelated among neurons whereas for *λ* > 0 and *λ* < 0 internal noise is positively and negatively correlated between the nearest neighbor neurons, respectively. The common input is a harmonically modulated signal *S*(*t*) = *A*cos(*ωt*), *A <* 1.

Given that the response of each neuron is not affected by the correlation between the spatially nearest neighbor neurons, it is important to calculate the spectral statistics of the array of LIF neurons. Introducing the Fourier transforms of the zero average spike trains [17]

$$y_{i}(\omega )=\frac{1}{\sqrt{T}}\int_{0}^{T}dt\exp(i\omega t)(y_{i}(t)-r_{0}(D)),$$ (4)

where *r*_0_(*D*)denotes the steady firing rate of a single neuron in the noise level *D*. The summed output response *y*(*t*) will be a cyclostationary random signal with the same period *T* = *2π/ω* since the weak signal *S*(*t*) is periodic. Finally, ensemble response *y*(*t*) is defined as the average of output spikes given by

$$y(t)=\frac{1}{N}\sum_{i=1}^{N}y_{i}(t).$$ (5)

### SNR gain

The LIF model has been used to investigate the transmission of signals polluted by external noise and under the presence of independent internal noise in neural ensembles [25, 26]. As a result, both noise sources have been shown to induce SR and efficient high-frequency transmission. In addition, recent research has focused on the analysis of neural ensembles considering noise correlation [13, 15, 36–39]. Accordingly, we focus on theoretical analysis and simulations to study SR and SNR gain in a LIF neural ensemble by including the correlation of noise. Hence, this study represents an extension to previous research, e.g., [25, 26].

We assume that the external input signal *S*(*t*) is weak, i.e., that the standard deviation of this term is small compared to the other terms in Eq (2). In particular, this periodic stimulus cannot elicit a spike. Weak signals obey the linear response theory; hence, each LIF neuron can be taken as a linear filter, with its frequency-domain linear response given by [17]

$$y_{i}(\omega )=y_{i,0}(\omega ,D)+B(\omega ,D)S(\omega ),$$ (6)

where *y*_*i*,0_(*ω*) is the unperturbed term and *B*(*ω*,*D*) is the linear susceptibility at noise level *D*. We have assumed that both internal and external noises are white and Gaussian, the single neuron cannot distinguish between both kinds of noises. This assumption also extends to the firing rate *r*_0_(*D*) and the susceptibility function *B*(*ω*,*D*) that should be taken at noise intensity *D*. We can calculate respectively the firing rate and linear susceptibility *B*(*ω*,*D*) by the following expressions [25]

$$r_{0}(D)={\left[\tau_{r}+\sqrt{\pi }\int_{\left(\mu -V_{th}\right)/\sqrt{2D}}^{\left(\mu -V_{r}\right)/\sqrt{2D}}dze^{z^{2}}\mathrm{erfc}(z)\right]}^{-1},$$ (7)

$$B(\omega ,D)=\frac{r_{0}(D)i\omega }{\sqrt{D}(i\omega -1)}\frac{D_{i\omega -1}(\frac{\mu -V_{th}}{\sqrt{D}})-e^{\beta }D_{i\omega -1}(\frac{\mu -V_{r}}{\sqrt{D}})}{D_{i\omega }(\frac{\mu -V_{th}}{\sqrt{D}})-e^{i\omega \tau_{r}}e^{\beta }D_{i\omega }(\frac{\mu -V_{r}}{\sqrt{D}})},$$ (8)

where *β* = [*V*_*r*_^*2*^ *− V*_*th*_^*2*^ + 2*μ*(*V*_*th*_ *− V*_*r*_)]*/*4*D* and $D_{a}(z)$ is the parabolic cylinder function [26], which can be numerically obtained. Using this approximation, the power spectrum of the spike train for each neuron $P(\omega ,D)=\underset{T\to \infty }{\lim}\langle y_{i}(\omega )y_{i}^{*}(\omega )\rangle$ can be obtained as [25, 26]

$$P(\omega ,D)=P_{0}(\omega ,D)+{\left|B(\omega ,D)\right|}^{2}R_{SS}(\omega )$$ (9)

with

$$P_{0}(\omega ,D)=r_{0}(D)\frac{{\left|D_{i\omega }(\frac{\mu -V_{th}}{\sqrt{D}})\right|}^{2}-e^{2\beta }{\left|D_{i\omega }(\frac{\mu -V_{r}}{\sqrt{D}})\right|}^{2}}{{\left|D_{i\omega }(\frac{\mu -\nu_{th}}{\sqrt{D}})-e^{i\omega \tau_{r}}e^{\beta }D_{i\omega }(\frac{\mu -\nu_{r}}{\sqrt{D}})\right|}^{2}}$$ (10)

where *R*_*SS*_(*ω*) is the spectral density of the weak periodic signal.

The cross power spectrum between the *i*-th and (*i*+1)-th neurons can be obtained by decomposing the corresponding inputs:

$$I_{i}=\mu +\sqrt{2D(1-\left|\lambda \right|)}\xi_{i}(t)+[\sqrt{2D\left|\lambda \right|}\xi_{c}(t)+S(t)],$$ (11)

$$I_{i+1}(t)=\mu +\sqrt{2D(1-\left|\lambda \right|)}\xi_{i+1}(t)+[\sqrt{2D}\mathrm{sgn}(\lambda )\sqrt{\left|\lambda \right|}\xi_{c}(t)+S(t)],$$ (12)

where sgn(*x*) is the sign function. The internal noise terms *ξ*_*i*_(*t*) and *ξ*_*c*_(*t*) are specific for each or common to all neurons, respectively. All these noises are uncorrelated among each other. Note that because of the scaling by the factors $\sqrt{\left(1-\left|\lambda \right|\right)}$ and $\sqrt{\left|\lambda \right|}$ in Eqs (11) and (12) the total internal noise have a fixed intensity irrespective of the value of the correlation parameter *λ*. Considering the terms in square brackets of Eqs (11) and (12) as perturbation, an approximation of the cross power spectrum for two nearest neighbor neurons $P_{cross}(\omega )=\underset{T\to \infty }{\lim}\langle y_{i}(\omega )y_{{}_{i+1}}^{*}(\omega )\rangle$ can be written as

$$P_{cross}(\omega ,D)={\left|B(\omega ,D(1-\left|\lambda \right|))\right|}^{2}[2\lambda D+R_{ss}(\omega )].$$ (13)

We now turn to discuss the power spectrum of the neural ensemble (for general size *N*). The ensemble as a whole has been characterized by a collective output *y*(*t*) in Eq (5). The power spectrum for the neural ensemble can be given by

$$\begin{array}{c} P_{yy}(\omega ,D)=P_{cross}(\omega ,D)+\frac{1}{N}[P(\omega ,D)-P_{cross}(\omega ,D)]\\ \;=\frac{1}{N}P(\omega ,D)+\frac{N-1}{N}P_{cross}(\omega ,D) \end{array}.$$ (14)

It can found that the power spectrum for the neuron ensemble is dominated by the cross correlation between nearest neighbor output spike trains; and other contributions enter only 1/*N*. Separating the power spectrum of the spike train for each neuron into coherent and incoherent parts, we have

$$P^{coh}(\omega ,D)={\left|B(\omega ,D)\right|}^{2}R_{SS}(\omega ),$$ (15)

$$P^{incoh}(\omega ,D)=P_{0}(\omega ,D).$$ (16)

Separating the cross power spectrum into coherent and incoherent parts, we have

$$P_{cross}^{coh}(\omega ,D)={\left|B(\omega ,D(1-\left|\lambda \right|))\right|}^{2}R_{SS}(\omega ),$$ (17)

$$P_{cross}^{incoh}(\omega ,D)=2\lambda D{\left|B(\omega ,D(1-\left|\lambda \right|))\right|}^{2}.$$ (18)

Based on Eqs (14–18), the power spectrum of the neural ensemble is given by

$$\begin{array}{l} P_{yy}(\omega ,D)=\frac{1}{N}[P_{0}(\omega ,D)+{\left|B(\omega ,D)\right|}^{2}R_{ss}(\omega )]\\ \;+\frac{\left(N-1\right)}{N}{\left|B(\omega ,D(1-\left|\lambda \right|))\right|}^{2}R_{SS}(\omega )\\ \;+\frac{2\lambda D(N-1)}{N}{\left|B(\omega ,D(1-\left|\lambda \right|))\right|}^{2} \end{array}.$$ (19)

Next, we study the improvement of signal-to-noise ratio (SNR) induced by nearest neighbor correlated noise. For simplicity, we consider a weak periodic signal to be *S*(*t*) = *A*cos(*ωt*), *A <* 1, whose output SNR is given by

$$R_{out}=\frac{P_{yy}^{coh}(\omega ,D)}{P_{yy}^{incoh}(\omega ,D)},$$ (20)

with

$$P_{yy}^{coh}(\omega ,D)=\frac{1}{N}{\left|B(\omega ,D)\right|}^{2}R_{ss}(\omega )+\frac{\left(N-1\right)}{N}{\left|B(\omega ,D(1-\left|\lambda \right|))\right|}^{2}R_{SS}(\omega ),$$ (21)

$$P_{yy}^{incoh}(\omega ,D)=\frac{1}{N}P_{0}(\omega ,D)+\frac{2\lambda D(N-1)}{N}{\left|B(\omega ,D(1-\left|\lambda \right|))\right|}^{2}.$$ (22)

The theoretical expression of input SNR can be calculated by

$$R_{in}=\frac{\pi A^{2}}{4D}.$$ (23)

Then, from the definitions of output and input SNR, the SNR gain of the neural ensemble can be defined by

$$G=\frac{R_{out}}{R_{in}}.$$ (24)

Hence, Eqs (20) and (23) provide a generic expression to evaluate the SNR of neural ensembles. If SNR gain *G* exceeds unity, the interactions of the neurons in the ensemble and their controllable internal noise provide an improvement in the input signal.

## Results and discussion

The performance of single LIF neurons has been widely analyzed [4, 12–22]. In contrast, we mainly examine the collective response of an LIF neural ensemble. We evaluated the SR by analyzing the variation of the output SNR as a function of the intensity of internal noise. In addition, from the definition in Eq (24), we analyzed the evolution of *G* for different system parameters. If SNR gain *G* is above unity at the optimal internal noise level, we say that the internal noise of the neural ensemble improves or amplifies the weak periodic signal at the input.

In this paper, we also compare the analytical results to numerical simulations. We will analyze the output SNR and the SNR gain at different array sizes (*N* = 100, and *N* = 1000), internal noise intensity, and different values of the correlation parameter *λ*. The numerical simulations were obtained as follows. We used an Euler scheme with a time step Δ*t* = 10^−3^ for numerically integrating stochastic differential Eq (1). For each array size *N* and correlation coefficient *λ* we average the results of 1000 realizations of the noises and the signal. The power spectrum can be calculated as the average of 1000 such recordings. Thus, for the given power spectrum, the output SNR can be evaluated by

$$R_{out}=\frac{\mathrm{Power}\;\mathrm{spectrum}\;\mathrm{at}\;\mathrm{signal}\;\mathrm{frequency}}{\mathrm{Noise}\;\mathrm{power}\;\mathrm{spectrum}}.$$ (25)

**Fig 2:**
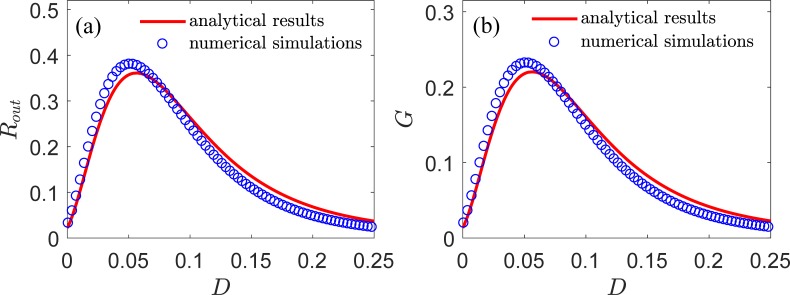
Output SNR and SNR gain for a single LIF neurons (*N* = 1). The analytical and numerical results are shown in lines and symbols, respectively. (a) Output SNR *R*_*out*_ and (b) SNR gain *G* according to the intensity *D* of internal noise for input *S*(*t*). The remaining parameters are *V*_th_ = 1, *V*_r_ = 0, *τ*_r_ = 0.1, *μ* = 0.8, *ω* = 0.1, and *A* = 0.5.

If the number of neurons, *N*, is 1 and response $y(t)=\frac{1}{N}\sum_{i}x_{i}(t)=x_{1}(t)$, the resulting system has a single LIF neuron and displays the conventional SR or residual SR [4, 12–22]. We selected *V*_*th*_ = 1, *V*_r_ = 0, *τ*_r_ = 0.1, *μ* = 0.8, *ω* = 0.1, and *A* = 0.5. We show in Fig 2 the variation of output SNR *R*_out_ and SNR gain *G* according to the intensity *D* of internal noise. The result of the output SNR *R*_out_ in Fig 2(A) confirms the phenomena of the conventional SR. The SNR gain *G* is below unity, as illustrated in Fig 2(B). The results of numerical simulations (represented by symbols in the figure) are also shown in Fig 2 and are in good agreement with the analytical results. These numerical results indicate that the present theoretical framework can fully describe the SR phenomena in a single LIF neuron. In the following, we will show the analytical results and numerical simulations in the LIF neural ensemble.

**Fig 3:**
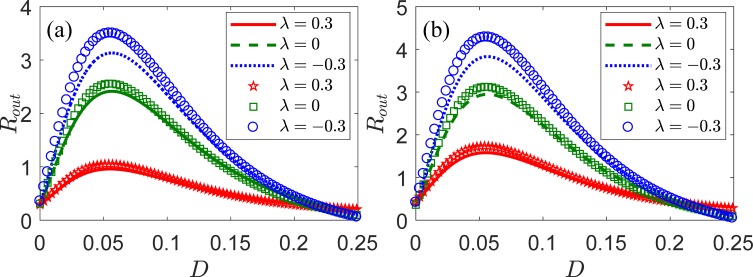
Output SNR *R*_out_ according to intensity *D* of internal noise for an ensemble of LIF neurons with different correlation coefficient *λ*. The analytical and numerical results are shown in lines and symbols, respectively. Positive (*λ* = 0.3, solid lines, pentagons), negative (*λ* = *−*0.3, dotted lines, circles), and independent (*λ* = 0, dashed lines, squares) correlations, and (a) *N* = 100, (b) *N* = 1,000 neurons are considered. The remaining parameters are *V*_th_ = 1, *V*_r_ = 0, *τ*_r_ = 0.1, *μ* = 0.8, *ω* = 0.1, and *A* = 0.5.

First, we analyze the dependence of output SNR *R*_out_ on the intensity *D* of internal noise with correlation coefficient *λ* for different *N* in the LIF neural ensemble. We selected *V*_th_ = 1, *V*_r_ = 0, *τ*_r_ = 0.1, *μ* = 0.8, *ω* = 0.1, and *A* = 0.5. Next, Fig 3(A) shows the variation of output SNR *R*_out_ according to intensity *D* of internal noise for different values of *λ* when *N* = 100. It can be seen a nonmonotonic evolution of the output SNR with respect to the intensity of internal noise, thus exhibiting typical SR characteristics and implying that the internal noise can improve the collective response of the neural ensemble for weak periodic signals. Furthermore, the intensity of internal noise shows an optimal value, where the output SNR reaches a maximum. In addition, the highest value of *R*_out_ is reached for a negative correlation, *λ* = *−*0.3. Hence, this correlation is the most suitable for activating neurons and improving the input signal among the three types of correlations, namely, independent (*λ* = 0), positive (*λ* = 0.3), and negative (*λ* = *−*0.3). Similarly, Fig 3(B) shows the variation of output SNR *R*_out_ according to the intensity *D* of internal noise for different values of *λ* when *N* = 1,000. Compared to Fig 3(A), it can be seen that the maximum output SNR increases with the number of the neurons.

**Fig 4:**
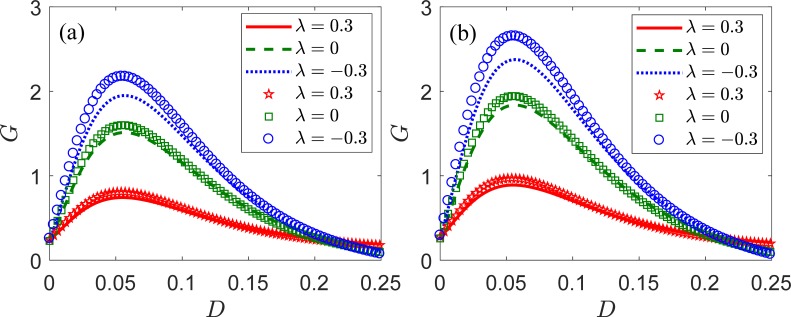
SNR gain *G* according to intensity *D* of internal noise for an ensemble of LIF neurons with different correlation coefficient *λ*. The analytical and numerical results are shown in lines and symbols, respectively. Positive (*λ* = 0.3, solid lines, pentagons), negative (*λ* = *−*0.3, dotted lines, circles), and independent (*λ* = 0, dashed lines, squares) correlations, and (a) *N* = 100, (b) *N* = 1,000 neurons are considered. The remaining parameters are *V*_th_ = 1, *V*_r_ = 0, *τ*_r_ = 0.1, *μ* = 0.8, *ω* = 0.1, and *A* = 0.5.

Fig 4 shows SNR gain *G* according to intensity *D* of internal noise for a fixed weak periodic signal, *S*(*t*), in an LIF neural ensemble with different correlation coefficient *λ*. It can be seen that the SNR gain also displays a nonmonotonic behavior with the increasing intensity of internal noise for the cases of statistical independence (*λ* = 0), positive (*λ* = 0.3) and negative correlation (*λ* = *−*0.3). These collective phenomena can be termed as array SR. In these cases, the SNR gain is higher than unity at the optimal intensities of internal noise, which is different from the conventional SR shown in Fig 2. Fig 4(A) and 4(B) show that the negative correlation assists the collective response of the neural ensemble to improve the signal, and the SNR gain is higher for an increasing number of the neurons. It is worth noting that the SNR gain does not reach unity for a positive correlation for *N* = 100. However, the SNR gain can be tuned to exceed unity for moderately large size of neuron array *N*, as illustrated in Fig 4(B).

We now explain why the output SNR and SNR gain can be enhanced by internal noises with negative nearest neighbor correlation. When an array of neurons acts in parallel, if one of the neurons makes a mistake (for example, fires when the signal is low or fails to fire), other neurons that are negatively correlated or independent with it are not likely to repeat the mistake. This effect is even stronger for the case that the other neurons are negatively correlated with the one that makes the mistake than for the case that other neurons are independent. In other words, nearest neighbor negative correlations between noises at different sites tend to repeat them. It is not going to lead to a visible increase in the spectrum peak at the signal frequency, but it may lead to a decrease of the noise background. According to the definition of output SNR in Eq (20), the cross power spectrum contribution 2*λD*|*B*(*ω*,*D*(1−|*λ*|))|^2^(*N*−1)/*N* to the incoherent part of the cross power spectrum in Eq (22) becomes negative value when the nearest neighbor correlation is negative. Thus, the denominator term of output SNR ($P_{yy}^{incoh}(\omega ,D(1-\left|\lambda \right|))$) is smaller for negative correlation than that for other types of correlations, regardless of other parameters. As a result, the output SNR and SNR gain can benefit from the negative nearest neighbor correlation of internal noises. Thus, the mechanism of enhancing the SNR relies on lowering the noise background, not on elevating the signal peak. In addition, we note that the output SNR and SNR gain increases significantly for all intensities of internal noise as the array size increases whether the nearest neighbor correlation is negative, positive or zero. It can be concluded that the enhanced SNR and SNR gain is not of any specific dynamics of neural array but is present also in arrays of uncoupled neurons. This, together with previous results observed in other systems [25,26], shows that the enhanced SR is a generic feature of arrays.

**Fig 5:**
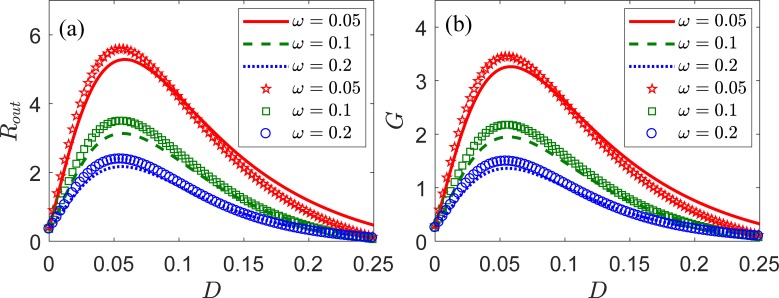
SNR and SNR gain for different frequencies. The analytical and numerical results are shown in lines and symbols, respectively. *ω* = 0.05 (solid lines, pentagons), *ω* = 0.1 (dashed lines, squares), and *ω* = 0.2 (dotted lines, circles). (a) Output SNR *R*_*out*_ and (b) SNR gain *G* according to the intensity *D* of internal noise for input *S*(*t*). The remaining parameters are *V*_th_ = 1, *V*_r_ = 0, *τ*_r_ = 0.1, *μ* = 0.8, *λ* = *−*0.3, *N* = 100, and *A* = 0.5.

The results above show that the highest SNR is achieved with a negative spatially nearest neighbor correlation of internal noise. However, these results held for a fixed angular frequency *ω* = 0.1. Hence, our next step was to study the evolution of output SNR *R*_out_ and SNR gain *G* according to the intensity *D* of internal noise with *λ* = *−*0.3 for different frequency values *ω*, and the results are shown in Fig 5(A) and 5(B) for the output SNR and SNR gain, respectively. Fig 5(A) shows that *R*_out_ increases with the decreasing of frequency, and the maximum *R*_out_ is reached at the lowest frequency, *ω* = 0.05. Nevertheless, the intensity of internal noise that corresponds to the maximum *R*_out_ is larger for the frequency of 0.05 than for the other two frequencies in the graph. Likewise, Fig 5(B) shows that the SNR gain also increases with the decreasing of frequency. Clearly, the SNR gain corresponding to the optimal intensity of internal noise is larger than unity for all the frequencies, i.e., *ω* = 0.05, 0.1, 0.2. Therefore, a SNR gain above unity can be considered as a general characteristic when using negative correlation in an LIF neural ensemble, regardless of the frequency. In addition, Fig 5(B) shows that the SNR gain at the optimal intensity of internal noise becomes larger as the input signal becomes slower. Therefore, we can conclude that low-frequency signals (e.g., *ω* = 0.05) in a noisy environment can notably induce the collective response of neural ensembles. It is important to understand mechanism responsible for the enhanced output SNR and gain. The shape of the low-frequency signal is resolved much better compared to the high-frequency signal. The low-frequency signal in noisy environment is more possible to be detected by the LIF neuron model. Thus, the output SNR and SNR gain increases as the decreasing of the signal frequency. The present results agree with observations in several nonlinear physical and biological systems [17, 40–42].

We have shown that the SNR gain depends on correlation coefficient *λ*, input signal frequency *ω*, and intensity *D* of internal noise. Next, we evaluated the effect caused by the amplitude of the weak periodic signal received by the neural ensemble. This effect is important because the sensory nervous system usually presents complex signals. Moreover, external stimuli of the sensory nervous system produces coding patterns determined by oscillations in the amplitude of calcium-ion activity. This activity is characterized by periodic signals that enter the nucleus to induce response variations [43–46], and several experimental and theoretical studies report the amplitude modulation of calcium-ion activity induced by external stimulus [44, 45].

**Fig 6:**
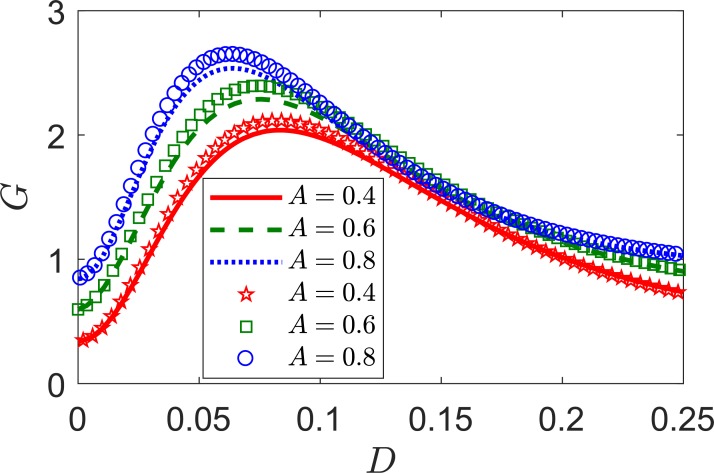
SNR gain for different signal amplitudes. The analytical and numerical results are shown in lines and symbols, respectively. *A* = 0.4 (solid lines, pentagons), *A* = 0.6 (dashed lines, squares), and *A* = 0.8 (dotted lines, circles). The remaining parameters are *V*_th_ = 1, *V*_r_ = 0, *τ*_r_ = 0.1, *μ* = 0.8, *λ* = *−*0.3, *ω* = 0.1, and *N* = 100.

In Eqs (21–24), the input and output SNR (*R*_in_ and *R*_out_) are determined by the weak signal amplitude (*A*), the internal noise intensities (*D*). Regardless the nearest neighbor correlation and frequency of weak signal, the signal amplitude could affect the input and output SNR. Accordingly, the SNR gain will also vary with *A*. By selecting different values of *A*, we calculated SNR gain *G* to verify the effect of a varying input signal amplitude *A*. Fig 6 show SNR gain *G* according to the intensity *D* of internal noise for amplitudes *A* = 0.4, 0.6, 0.8, respectively. The figure show increasing maximum SNR gain values as input signal amplitude *A* increases, with the highest SNR reached at *A* = 0.8. Thus, high amplitude of a weak signal (i.e., a signal with *A <* 1) induces a more pronounced SNR gain of the neural ensemble. In addition, Fig 6 show that the maximum SNR gain values for different amplitudes are located at the different optimal noise level *D*. Furthermore, the curves show SNR gain *G* larger than unity even for the smallest signal amplitude, *A* = 0.2, provided that the correlation is negative, i.e., *λ* = *−*0.3. Thus, we can conclude that the SNR gain can be improved by varying the amplitude of a weak periodic signal when considering a negative correlation. Hence, amplitude is another factor to improve signal processed by an LIF neural ensemble. It is worth noting that both intensity of noise and the amplitude of the weak periodic signal can be controlled in a wide range that can be tuned for specific signal processing applications [30].

## Conclusions

We investigated the SNR gain of a parallel array of LIF neurons that receives a weak periodic signal and presents spatially nearest neighbor correlated noise. By using linear response theory, we derived the analytic expressions of SNR and SNR gain. This article reports that the intensity of internal noise can be increased up to an optimal value, where the output SNR reaches its maximum. This property suitable for noise-enhanced transmission of weak periodic signals can be related to SR. In addition, the results show that the spatially nearest neighbor correlation of noise in an LIF neural ensemble can influence the collective response of neurons. In particular, the output SNR according to the intensity of internal noise presented a more pronounced peak when the spatially nearest neighbor correlation is negative, i.e., negative correlation has more beneficial effect that the other types, namely, positive correlation and independence, in the signal enhancement. The improvement of SNR gain resulting from noise in an array of elements can be related to the array SR [23].

We also found that the SNR gain can be further improved by tuning the number of neurons in the arrays and input signal frequency. Likewise, the region that SNR gain exceeds unity, which implies an improved signal, can be modulated by tuning some parameters. Our investigation showed that increasing the number of neurons in the array induces high SNR gains regardless of the correlation type, thus improving the signal qualities for transmission. In addition, low-frequency periodic signals induce higher SNR gains than high-frequency signals, which might allow to explain the common sensitivity of the brain to low-frequency and discrete weak periodic signals [40–42]. In fact, the brain has cells have fast and slow adaptation mechanisms, which react in periods ranging from less than a millisecond to tens of milliseconds.

Another source of SNR gain improvement is the variation in the amplitude of the weak periodic signal, and we evaluated the SNR gain with more realistic signal inputs that involve modulated amplitude. The corresponding results might provide insights on the spontaneous generation of spike trains in the auditory systems, which are also related to SR. Although model and experiments should be further studied to optimize the parameters, existing research in biophysical phenomena [40–42] supports our results on the benefits of internal noise to improve signals. Overall, this article illustrates the potential to utilize spatially nearest neighbor correlated noise and the number of neurons in an ensemble for improving the collective response to inputs, and provides guidelines for designing signal processing methods to transmit improved weak periodic signals.
